# Autologous Cell‐Free Fat Extract: A Novel Approach for Infraorbital Rejuvenation—A Pilot Study

**DOI:** 10.1111/jocd.16682

**Published:** 2024-12-08

**Authors:** Yizuo Cai, Zhuoxuan Jia, Jiancheng Gu, Bijun Kang, Wei Li, Wenjie Zhang

**Affiliations:** ^1^ Department of Plastic and Reconstructive Surgery, Shanghai 9th People's Hospital, Shanghai Jiao Tong University School of Medicine, Shanghai Key Laboratory of Tissue Engineering National Tissue Engineering Center of China Shanghai China; ^2^ Division of Plastic Surgery Shanghai Basilica Clinic Shanghai China

**Keywords:** aesthetic medicine, aging face, growth factors, rejuvenation, stem cells

## Abstract

**Background and Objective:**

CEFFE (Cell‐free fat extract) treatment for periocular fine lines requires thorough clinical evaluation to determine its efficacy and safety in enhancing skin quality. The research enrolled 10 healthy female participants aged 31–58, focusing on skin texture, elasticity, and barrier function.

**Methods and Results:**

CEFFE treatment demonstrated significant benefits, with notable improvements observed as early as 3 months posttreatment, which continued throughout the 12‐month follow‐up period. Objective assessments revealed reductions in SEr% and SEw%, indicative of reduced skin roughness and wrinkles, particularly pronounced after the third month of treatment. Enhanced skin elasticity, as indicated by improvements in R2%, R5%, and R7%, was observed, with the most significant enhancements noted at the 6‐month follow‐up. Furthermore, TEWL decreased consistently, highlighting CEFFE's potential in maintaining the skin's barrier function and moisture retention. High patient satisfaction levels, with 70% expressing satisfaction ranging from satisfied to very satisfied, underscored CEFFE's clinical significance.

**Conclusions:**

CEFFE demonstrates potential as an effective and safe intervention for addressing periocular fine lines, providing a solution for fine lines while ensuring skin health (ChiCTR1900024329).

## 1 Introduction

Skin aging, characterized primarily by the progressive loss of collagen, presents a distinctive manifestation in the infraorbital region, particularly evident in the lower eyelids, resulting in diminished elasticity, heightened pigmentation, and the emergence of unsightly wrinkles [1]. While skin aging is an inexorable natural phenomenon, its deleterious effects can be ameliorated through an array of therapeutic interventions [1, 2].

Numerous strategies have been advanced in the ongoing battle against skin aging, encompassing the utilization of antioxidants, platelet‐rich plasma (PRP), and stromal vascular fraction (SVF) [3, 4, 5]. However, the effectiveness of these modalities exhibits significant variability. For example, while topical antioxidants offer convenience, their efficacy is often constrained by the formidable barrier presented by the skin itself [3, 4, 5]. Conversely, the intradermal administration of PRP and SVF has garnered increasing attention [6, 7]. Nevertheless, the efficacy of these approaches remains a subject of debate, with PRP efficacy influenced by the variability in platelet concentrations and SVF hindered by its intricate and costly preparation process [8, 9].

A novel adipose‐derived derivative, denominated as cell‐free fat extract (CEFFE), is methodically isolated through additional centrifugation of adipose tissues, effectively segregating the oil and cellular fractions [10]. CEFFE is imbued with a diverse array of growth factors, comprising proangiogenic factors such as VEGF, bFGF, and PDGF, in addition to pro‐proliferative factors like TGF‐β and EGF [11, 12]. Encouragingly, reports have surfaced detailing the augmentation of dermal thickness following intradermal CEFFE injections, observed in both normal and photoaged skin in nude mice [13, 14]. This suggests the potential for skin rejuvenation through the facilitation of neovascularization and enhanced collagen production. Consequently, the principal objective of this study was to meticulously assess the efficacy and safety profile of CEFFE in addressing infraorbital aging.

## 2 Methods

### 2.1 Subjects, Inclusion and Exclusion Criteria

This preliminary report has been approved by the Committee on Clinical Investigation of the Shanghai Ninth People's Hospital (IRB, SH9H‐2019‐T135‐3) and registered at chictr.org.cn (ChiCTR1900024329). In accordance with the Declaration of Helsinki, all 10 enrolled patients, displaying initial signs of infraorbital aging, provided informed consent prior to participation between August 2019 and December 2019.

The inclusion criteria were as follows: (1) History of infraorbital aging; (2) Wrinkle severity rating scale (WSRS) ≥ 2 (1, no visible fold; 2, shallow but visible folds, minor facial feature; 3, moderately deep folds, a clear facial feature visible at normal appearance but not when stretched; 4, very long and deep folds, prominent facial feature, < 2 mm folds when stretched; and 5, extremely deep and long folds, detrimental to facial appearance, 2–4 mm V‐shape folds when stretched); (3) Age between 30 and 65; (4) body mass index (BMI) between 20 and 29.9.

The exclusion criteria included: (1) Ulceration surrounding the lower eyelid; (2) pregnancy; (3) unrealistic patient expectations; (4) severe mental disorders; (5) blood‐borne diseases; (6) lidocaine allergy history; (7) participation in any other research project/clinical trial. Patient data, including age, sex, weight, height, and BMI, were recorded.

### 2.2 CEFFE Preparation and Treatment

All patients underwent adipose tissue aspiration to prepare CEFFE [10]. Liposuction was performed using a standard 3 mm cannula with large side holes (2 × 7 mm) after infiltration with a modified Klein solution containing 800 mg/L lidocaine and a 1:1 000 000‐unit dilution of adrenaline into the abdomen. Approximately 100 mL of fat was collected from each patient. The harvested fat tissues were rinsed with saline solution to remove residual blood cells and then centrifuged at 1200 *g* for 3 min. The middle fat layer was retained for further emulsification, while the other layers were discarded. The fat tissue was connected by a 2 mm Luer lock connector (B.BraunMedical Inc. Germany) and agitated 60 times. The emulsified fat was subsequently centrifuged at 1200 *g* for 5 min and separated into four layers (oil, fat, CEFFE, and cell fragment pellet). CEFFE was collected and filtered through a 0.22‐μm filter (Corning, USA) and stored at −20°C in a 1.8‐mL sterilized cryopreservation tube (Thermo Fisher Scientific, USA). Approximately 10 mL of CEFFE was obtained from each patient. The specific preparation process can be found in Video S1.

**FIGURE 1:**
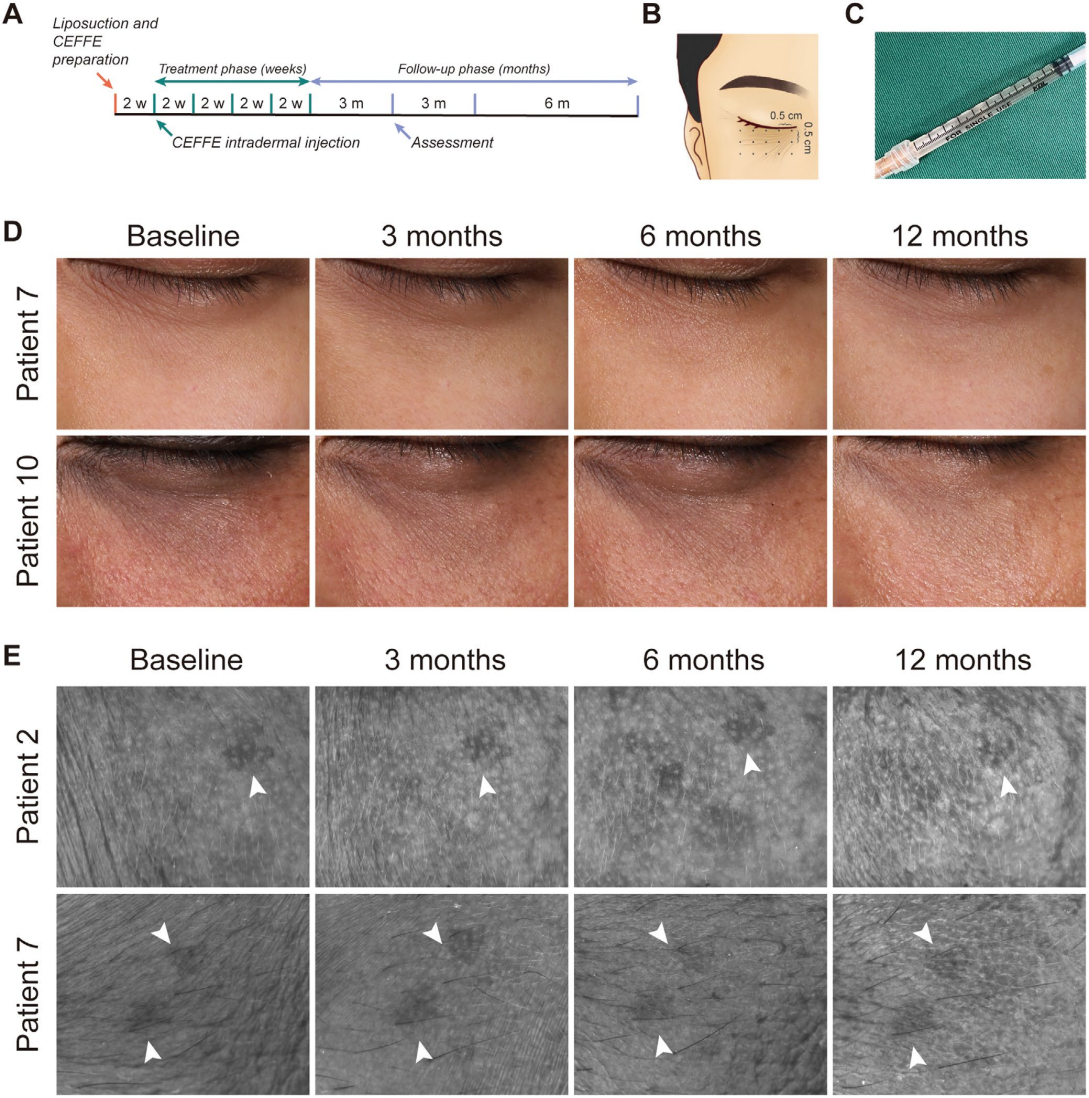
(A) Experimental workflow. (B) A diagram of the injection sites. (C) The state of CEFFE in a 1 mL syringe. (D) Representative infraorbital 2D images (Patient 7 and Patient 10). (E) Representative skin texture images (Patient 2 and Patient 7), with white arrows indicating infraorbital pigmentation as positioning markers.

Each patient received a series of five CEFFE infraorbital injections at 2‐week intervals, with the first injection administered 2 weeks after CEFFE preparation. Following the final infraorbital injection, patients underwent three follow‐up assessments at 3, 6, and 12 months posttreatment (Figure 1A).

One hour before each injection, both sides of the infraorbital areas were cleansed and topically anesthetized with an ointment comprising 25% lidocaine and 25% prilocaine (Ziguang, China). After disinfection, 1.8 mL of CEFFE was intradermally injected using a 32‐G needle by the nappage technique, with 0.05 mL of CEFFE per point in a linear pattern spaced 0.5 cm apart (Figure 1B,C).

### 2.3 Objective Efficacy Assessment

Standard two‐dimensional (2D) photographs were captured using VISIA‐CR (Canfield, USA). Skin texture was assessed using Vivoscan VC98 USB (Courage + Khazaka Electronic GmbH, Germany) [15, 16]. The testing points were fixed at the inner, middle, and lateral infraorbital areas for each patient. Skin texture was defined by the parameters SEr (roughness), SEsm (smoothness), and SEw (wrinkles). Skin elasticity was evaluated using Cutometer Dual MPA580 Elasticity Cutometer Probe (Courage + Khazaja Electronic GmbH, Germany) [4]. Elasticity was defined by the parameters *R*2 (ratio of elastic extension), *R*5 (net elasticity), and *R*7 (ratio of elastic recovery to total deformation). Skin barrier was evaluated using Cutometer dual MPA580 Tewameter TM300 Probe and defined in terms of transepidermal water loss (TEWL). All these measures were evaluated before treatment (baseline) and during each follow‐up visit (at 3, 6, and 12 months) after the last treatment. The change in these measures was calculated as follows:

$${\text{Measurements}}^{*}\%={\text{Measurements}}^{*}t/{\text{Measurements}}^{*}\text{baseline}\times 100\%$$

where Measurements * baseline represents the baseline (before CEFFE treatment) and Measurements * *t* represents data at 3, 6, or 12 months after the final treatment (*t* = 3, 6, or 12).

### 2.4 Subjective Evaluation

In comparison to baseline photographs, live assessments using the Global Aesthetic Improvement Scale (GAIS), a 5‐point scale ranging from “worse” to “very much improved,” were conducted by three independent medical professionals as well as self‐assessment by the patients during the 12‐month follow‐up visit [17]. Patient satisfaction at the 12‐month mark following the final treatment was evaluated using the Likert Satisfaction Scale (LSS). The LSS gauges specific items using a five‐point scale, ranging from “very dissatisfied” to “very satisfied” [18].

### 2.5 Statistical Analysis

Paired *t*‐test and Wilcoxon tests were used for comparisons. All analyses were performed using SPSS 13.0. *p* < 0.05 was considered statistically significant.

## 3 Results

### 3.1 Clinical Characteristics

**FIGURE 2:**
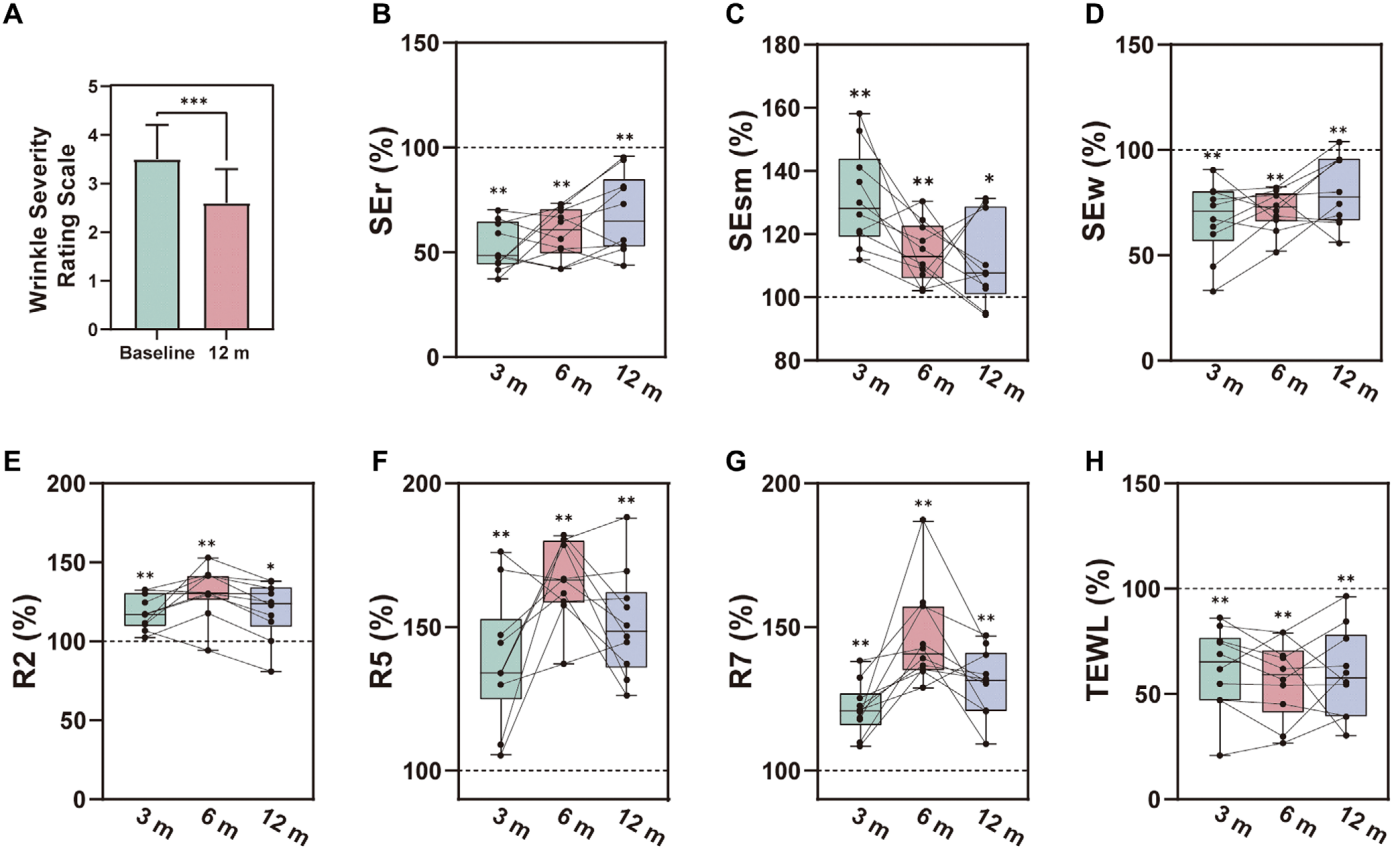
(A) Changes in WSRS between baseline and the 12‐month follow‐up. (B–D) Skin wrinkle improvement assessments at the 3‐, 6‐, and 12‐month follow‐ups, including SEr%, SEsm%, and SEw%. (E–G) Skin elasticity improvement assessments at the 3‐, 6‐, and 12‐month follow‐ups, including R2%, R5%, and R7%. (H) TEWL assessments at the 3‐, 6‐, and 12‐month follow‐ups.

Ten patients successfully completed all the follow‐up assessments. These individuals were healthy females with an age range of 31–58 years (44.60 ± 7.80). Their average height, weight, and body mass index (BMI) were 164.60 ± 2.58 cm, 61.45 ± 3.44 kg, and 22.67 ± 0.88 kg/m^2^, respectively (Table 1). Prior to treatment, the mean WSRS scores for all enrolled patients were 3.50 ± 0.67 (Figure 2A).

**TABLE 1:** Clinical characteristics of all 10 patients.

Patient no.	Sex	Age (years)	Height (cm)	Weight (kg)	BMI (kg/m^2^)
1	F	31	165	59.5	21.85
2	F	40	162	58	22.10
3	F	45	168	68	24.09
4	F	55	166	62	22.50
5	F	38	165	63	23.14
6	F	46	165	60	22.04
7	F	42	168	67	23.74
8	F	51	163	57	21.45
9	F	58	159	60	23.73
10	F	40	165	60	22.04

Abbreviation: F, female.

### 3.2 Objective Evaluation

Compared with the initial 2D standard photograph, varying degrees of improvement were noticed starting from the third CEFFE injection (Figure 1D and Figure S1). As time passed, these improvements became increasingly evident. These changes remained noticeable for a full 12 months, with the most significant improvement observed after 6 months of treatment. Fine lines and minor wrinkles in the infraorbital area became notably smoother, shallower, and in some cases even disappeared entirely. Concurrently, the skin exhibited a smoother and finer texture (Figure 1E).

SEr% and Sew% were positively correlated with skin roughness, while SEsm% showed a negative correlation with skin roughness. Over the three follow‐up assessments spanning 12 months, there were statistically significant differences (*p* < 0.05) in SEr%, SEsm%, and SEw% when compared to the baseline measurements, and individual trends were relatively consistent. Particularly at the 3‐month follow‐up, the most noticeable improvement in skin roughness was observed, with median values of 48.17%, 128.1%, and 70.41% for SEr%, SEsm%, and SEw%, respectively (Figure 2B–D).

Skin elasticity was assessed using R2%, R5%, and R7%. Over the 12‐month follow‐up period, there were statistically significant differences (*p* < 0.05) in R2%, R5%, and R7% compared with baseline measurements. The trends in R2% and R7% were relatively consistent among individuals, while R5% exhibited more noticeable individual variations. Particularly at the 6‐month follow‐up, the most prominent improvement in skin elasticity was observed, with median values of 117.10%, 133.90%, and 120.80% for R2%, R5%, and R7%, respectively (Figure 2E–G).

TEWL showed a significant decrease in all three follow‐up assessments (*p* < 0.05), with no significant changes observed with the extension of the follow‐up experiment. The median values for TEWL at the three follow‐up time points were 65.40%, 59.34%, and 57.85%, respectively (Figure 2H).

### 3.3 Subjective Evaluation

At 12‐month posttreatment, WSRS scores for all enrolled patients were 2.60 ± 0.67, showing a statistically significant difference compared to baseline (*p* < 0.05) (Figure 2A). Physician‐assessed GAIS scores averaged 3.60 ± 0.35, while patient self‐assessed GAIS scores averaged 3.70 ± 0.48. LSS score for all included patients was 4.00 ± 0.67. Furthermore, 70% of the patients (*n* = 7) expressed satisfaction ranging from satisfied to very satisfied with the CEFFE treatment (Table 2).

**TABLE 2:** GAIS and LSS.

Patient no.	Global Aesthetic Improvement Scale (GAIS)		Likert Satisfaction Scale (LSS)
	Physician (mean)	Patient	
1	3.33	4	4
2	3.67	4	4
3	3.00	3	3
4	3.33	3	3
5	3.67	4	5
6	3.33	4	4
7	3.67	3	4
8	4	4	5
9	4	4	4
10	4	4	4

### 3.4 Adverse Effects

All patients suffered transient and tolerable pain during the injection. Moreover, 50% of the patients (*n* = 5) showed mild bruises after the injection and recovered in 3.20 ± 2.5 days. One patient showed mild erythema that was relieved within 1 day. No other complications were observed in this study (Table 3).

**TABLE 3:** Adverse effects results.

Parameter	Number (%)	During (mean ± SD, days)
Bruise	5 (50%)	3.20 ± 2.50
Erythema	0	0
Edema	0	0
Burning	0	0
Stinging	10 (100%)	0.005 ± 0.003
Itching	0	0

## 4 Discussion

Our study provides evidence that CEFFE treatment yields substantial benefits in terms of skin quality, including improvements in skin texture, elasticity, and barrier function. Notably, these improvements were observed as early as 3 months after the initial treatment and persisted for the entire 12‐month follow‐up period.

The reduction in SEr%, SEsm%, and SEw% over the three follow‐up assessments indicates that CEFFE treatment effectively reduces skin roughness and wrinkles, leading to smoother and flatter skin. These improvements were particularly pronounced after the third month of treatment (Figure 2B–D). CEFFE's rapid impact on skin texture is promising for individuals seeking quick results. Skin elasticity is a fundamental aspect of youthful skin, and our study demonstrates that CEFFE enhances it effectively. The improvements in R2%, R5%, and R7% indicate increased skin elasticity, with the most significant enhancements observed at the 6‐month follow‐up (Figure 2E–G). CEFFE not only addresses existing wrinkles but also promotes long‐term skin health by improving elasticity. A significant reduction in TEWL was observed throughout the study, highlighting CEFFE's potential in restoring and maintaining the skin's natural barrier function and moisture retention. This effect can benefit individuals with infraorbital aging and other skin concerns related to moisture loss (Figure 2H).

The high level of patient satisfaction, with 70% expressing satisfaction ranging from satisfied to very satisfied, underscores the clinical significance of CEFFE treatment. The combination of objective improvements and patient‐reported satisfaction indicates the potential of CEFFE to improve patients' quality of life and self‐esteem (Table 2).

Despite these promising findings, several limitations should be noted. Our study had a relatively small sample size, consisting of 10 female participants, which limits the generalizability of the results. Additionally, the follow‐up period was limited to 12 months and long‐term assessments are needed to determine the durability of CEFFE treatment effects and whether maintenance treatments are required.

Furthermore, our study focused exclusively on female participants within a specific age range. Future research should investigate the effectiveness of CEFFE in addressing infraorbital aging in male patients and those outside this age range.

In clinical practice, hyaluronic acid (HA)‐based fillers have been proven safe and effective for rejuvenating the under‐eye area [19]. However, HA rapidly degrades in the body, and the delayed inflammatory responses that may arise from HA‐based fillers can lead to lower patient satisfaction, with minimal improvements in skin texture [20]. Recently, fat grafting and its derivatives, such as Coleman fat autologous fat transfer and nanofat, have shown potential for treating periorbital rejuvenation [21, 22, 23].

Coleman fat injections provide effective tissue filling while also improving skin quality [24]. However, their application around the eyes is somewhat limited due to particle size constraints. Additionally, subcutaneous injections may result in pale yellow discoloration and nodules [25]. There was study using sharp‐needle intrafermal fat grafting technology that may reduce nodules, fat necrosis, and cysts, though larger retrospective and prospective studies are needed to confirm these effects [26]. Nanofat is rich in a high concentration of stromal vascular cells, as well as various cytokines, growth factors, and biopeptides, which contribute to extracellular matrix remodeling, angiogenesis, and immune system regulation [27]. However, previous studies have indicated that nanofat may contain significant amounts of swelling liquid and oil, which could lead to inflammation and fibrosis postfilling [28].

Compared with these fat derivatives, CEFFE undergoes additional mechanical processing to remove cellular and oily components, enhancing safety, and reducing regulatory burdens. Furthermore, CEFFE's liquid form facilitates preparation and storage for clinical use. However, in treating under‐eye hollows, it may still be necessary to combine CEFFE with other therapies, such as hyaluronic acid. At the same time, the injection technique may also require further experimentation [29].

In conclusion, our study highlights the potential of CEFFE as an effective and safe intervention for infraorbital rejuvenation. CEFFE demonstrates remarkable improvements in skin quality, including texture, elasticity, and barrier function, with high patient satisfaction. Further research with larger sample sizes and prospective, randomized‐controlled designs is warranted to validate these findings and establish long‐term effects. CEFFE holds promise as a valuable addition to the armamentarium of aesthetic treatments, offering patients effective solutions for addressing infraorbital aging concerns while maintaining skin health and safety.

## Author Contributions

The study was designed by W.L. and W.Z. Y.C. and J.G. contributed to experimentation, data collection, and data analyses. Z.J. contributed to manuscript writing, editing, and data visualization. B.K. contributed to manuscript reviewed. All authors read and approved the final manuscript.

## Ethics Statement

This preliminary report has been approved by the Committee on Clinical Investigation of the Shanghai Ninth People's Hospital (IRB, SH9H‐2019‐T135‐3) and registered at chictr.org.cn (ChiCTR1900024329).

## Consent

The patients in this manuscript have given written informed consent to publication of their case details.

## Conflicts of Interest

The authors declare no conflicts of interest.

## Supporting information


**Video S1.** Video outlining the standardized protocol for CEFFE preparation.

**Figure S1.** Infraorbital 2D images for 10 patients were taken at baseline, and at 3‐, 6‐, and 12‐months post‐treatment.

## Data Availability

The data that support the findings of this study are available from the corresponding author upon reasonable request.
