# Supplementary material for: Autologous Cell‐Free Fat Extract: A Novel Approach for Infraorbital Rejuvenation—A Pilot Study
Source: J Cosmet Dermatol. 2024 Dec 8;24(2):e16682. doi: 10.1111/jocd.16682 (PMC11845974; doi:10.1111/jocd.16682)
Supplement: Supplementary file 2 — Figure S1. Infraorbital 2D images for 10 patients were taken at baseline, and at 3‐, 6‐, and 12‐months post‐treatment. [file JOCD-24-e16682-s002.docx]

Video S1. Video outlining the standardized protocol for CEFFE preparation


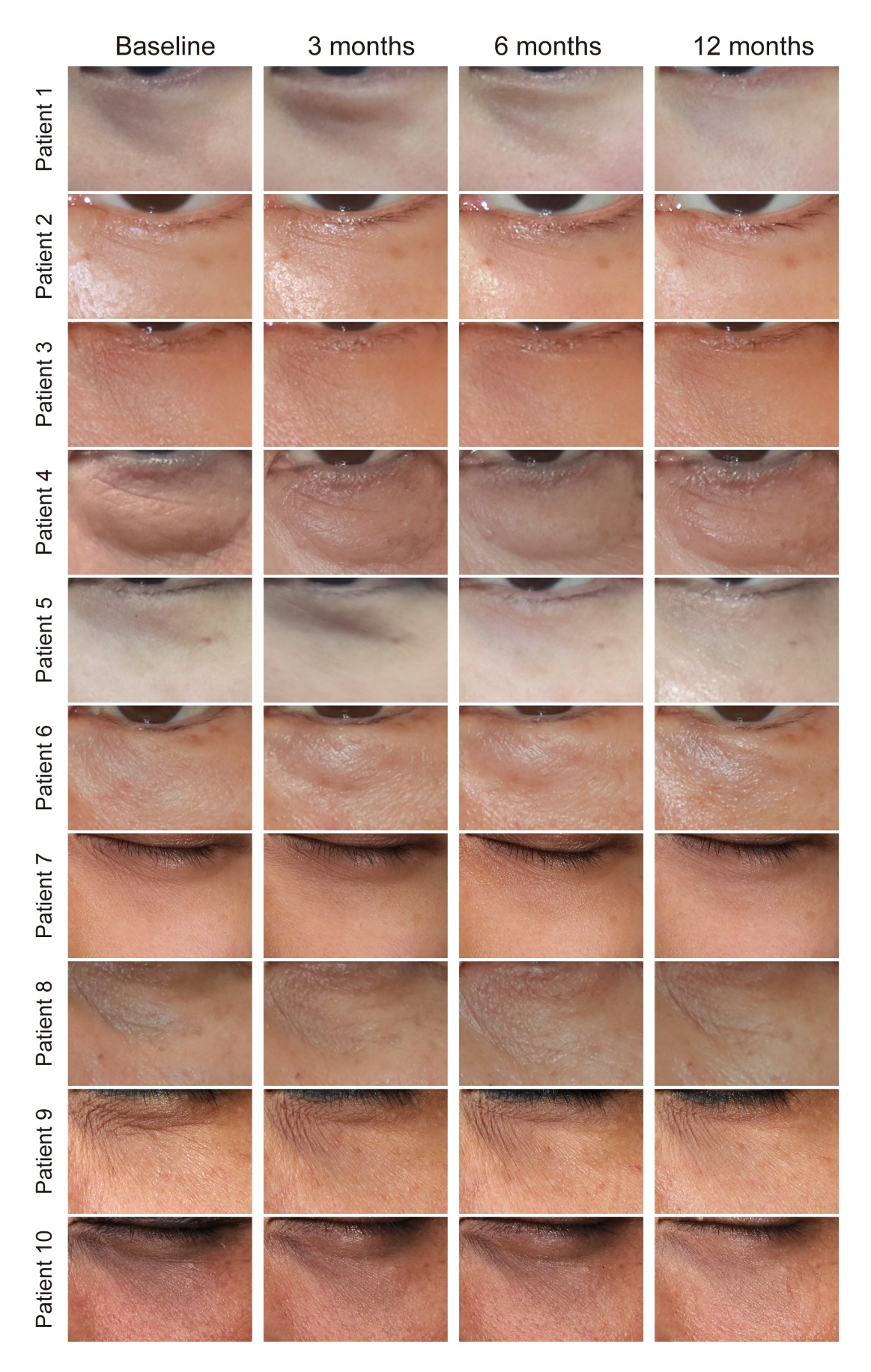
Figure S1: Infraorbital 2D images for 10 patients were taken at baseline, and at 3-, 6-, and 12-months post-treatment.
